# Metabolic syndrome and insulin resistance in pre-pubertal children with psoriasis

**DOI:** 10.1007/s00431-020-03924-w

**Published:** 2021-01-22

**Authors:** Francesca Caroppo, Alfonso Galderisi, Laura Ventura, Anna Belloni Fortina

**Affiliations:** 1 Pediatric Dermatology Unit – Department of Medicine DIMED, University of Padova, Via Gallucci, 4, 35128 Padova, Italy; 2 Department of Woman and Child’s Health, University of Padova, Padova, Italy; 3 Department of Statistics, University of Padova, Padova, Italy

**Keywords:** Pediatric psoriasis, Childhood psoriasis, Metabolic syndrome, Insulin resistance, HOMA-IR, Psoriasis

## Abstract

Psoriasis in adults is associated with an increased risk of metabolic disease. Various cardiometabolic comorbidities have been reported in childhood psoriasis, but only a few studies have analyzed the prevalence of metabolic syndrome. We performed a single-center prospective study investigating the prevalence of metabolic syndrome and insulin resistance in children with psoriasis. The prevalence of metabolic syndrome was evaluated in 60 pre-pubertal children with psoriasis (age: 3–10 years), accordingly to recently established criteria for the diagnosis of metabolic syndrome in children. Insulin resistance was considered altered when the homeostatic model assessment (HOMA-IR) for insulin resistance was ≥ 90th sex- and age-specific percentile and HOMA 2-IR was > 1.8. Eighteen (30%) children with psoriasis were found to have metabolic syndrome. Sixteen (27%) children were found to have insulin resistance.

*Conclusion*: Our data underline the importance of assessing metabolic syndrome not only in adults and adolescents but also in young children with psoriasis.

**Table:** 

**What is Known:** • *Psoriasis in adults is strongly associated with metabolic disease and insulin resistance.* • *Very limited data are available on the prevalence of metabolic syndrome and insulin resistance in pre-pubertal children with psoriasis*.	
**What is New:** • *This study reports that in pre-pubertal children with psoriasis, there is a high prevalence of metabolic syndrome and insulin resistance.* • *In children with psoriasis metabolic syndrome risk factors should be assessed*.

**Supplementary Information:**

The online version contains supplementary material available at 10.1007/s00431-020-03924-w.

## Introduction

Psoriasis in adults is associated with an increased risk of metabolic syndrome (MetS) [1–4].

Few data are available about this topic in children with psoriasis as most of the available studies analyze the prevalence of only single components of MetS [5–9] and not the overall prevalence of MetS [9].

The few studies on MetS in childhood psoriasis evaluated only a small number of children and a still more limited number of young children [10–12].

We performed a single-center study, investigating the prevalence of MetS and levels of insulin resistance in a population of pre-pubertal children with psoriasis and the correlations with clinical and anamnestic data.

## Methods

From 2014 to 2018, a prospective, single-center study of 60 consecutive pre-pubertal children with psoriasis (age between 3 and 10 years; 30 boys) was performed at the outpatient clinic of our tertiary center (Pediatric Dermatology Unit, Padua University, Padua, Italy).

The inclusion criteria of the study were children aged under 10 years with clinical diagnosis of psoriasis and with no other comorbidities reported.

The exclusion criteria were children taking any type of drugs, including systemic therapies for psoriasis.

The following data were collected for each patient by the same team of dermatologists experienced in pediatric dermatology: age, sex, height, body weight, body mass index (BMI), systolic and diastolic blood pressure (BP), waist circumference (WC), waist-to-height ratio (WHtR), and family history (first degree relatives affected) of psoriasis and metabolic or cardiovascular diseases (hypertension, diabetes mellitus, hypercholesterolemia).

The measurement of systolic and diastolic BP was performed using an aneroid sphygmomanometer with appropriately sized cuff for each patient. BP was measured in the right arm by using standard measurement practices.

BP was measured three times waiting 5 min between one measurement and another, and then the mean value was considered [13].

BMI calculated as weight in kilograms divided by height squared in meters allowed us to classify children as normal weight (< 85th and > 5th percentile), overweight (≥ 85th and < 95th percentile), and obese (≥ 95th percentile) [14].

Evaluation of central obesity was performed using WHtR, calculated as WC (in cm) divided by height (in cm). Patients with a WHtR ≥ 0.5 were classified as having central obesity [6].

Psoriasis severity was classified as mild or moderate-to-severe according to the Psoriasis Area Severity Index (PASI) and body surface area (BSA).

When PASI was ≥ 10 or/and BSA was > 10, psoriasis was designated as moderate-to-severe [6].

Triglycerides, high-density lipoprotein cholesterol (HDL-C), low-density lipoprotein cholesterol (LDL-C), total cholesterol (TC), insulin, and fasting blood glucose were measured in each patient using a Roche automated modular analyzer COBAS 8000 [15].

The prevalence of MetS in our group of children with psoriasis was evaluated and compared with the prevalence of MetS reported in a large European pediatric population (2–10 years) (“Identification and prevention of Dietary- and lifestyle-induced health Effects in Children and infantS”—IDEFICS study) [16].

The IDEFICS study, analyzing sex- and age-specific percentiles for each component of MetS (excess adiposity, high blood pressure, low levels of HDL-C, elevated triglycerides and glucose), identifies two levels of MetS in children: “monitoring level” (suggesting close monitoring of the child) and “action level” (suggesting the introduction of appropriate pediatric intervention), when the values of at least three of the five components are altered. WC, BP, triglycerides, and fasting blood glucose are considered altered with levels ≥ 90th sex- and age-specific percentile; HDL-C was considered altered with levels ≤ 10th sex- and age-specific percentile [16].

Levels of LDL-C and TC were evaluated according to sex- and age-specific reference values of blood lipid levels in European children aged 2.0–10.9 years. LDL-C and TC were considered altered with levels ≥ 90th sex- and age-specific percentile [17].

The homeostatic model assessment for insulin resistance (HOMA-IR) was calculated in each patient as [fasting glucose (mg/dl) × fasting insulin (lU/ml)/405] [18].

HOMA-IR was considered altered with levels ≥ 90th sex- and age-specific percentile according to normative values of HOMA-IR age- and gender-specific identified in a large population of Italian children [19].

The updated computer model of homeostatic assessment for insulin resistance (HOMA 2-IR) was also evaluated in each patient, using HOMA 2 Calculator V.2.2.3 [20, 21]. HOMA 2-IR > 1.8 was considered altered [22].

Oral consent was obtained from all children and their parents.

### Statistical analysis

Categorical variables were expressed as percentages and numerical variables as means (and standard deviations).

The χ^2^ test and Fisher’s exact test were utilized for the study of the association of categorical variables. The exact binomial test was used to test hypotheses on prevalence. Differences in quantitative variables between subject groups were assessed with Student *t* test or Mann-Whitney nonparametric test, according to the Shapiro-Wilk test of normality. Multiple penalized logistic regression [23] was used to model the two categories of children with and without MetS according to covariates not included in the definition of MetS (BMI, WHtR, normal weight, overweight, obesity, familial history of psoriasis, hypertension, diabetes mellitus, and hypercholesterolemia) using a backward model selection procedure. Statistical significance was defined as *p* < 0.05. Data analyses were performed using the free-software R (https://www.r-project.org).

The sample of the study included children with psoriasis referred to our center and sample size of the study allows to reach a power of 0.90, with an alpha = 0.05 and with a medium Cohen’s h effect size for proportions.

## Results

Demographic and clinical data of patients are shown in Table 1.

**Table 1:** Demographic and clinical data of patients

	Children with and without MetS (*n* = 60)	Children without MetS (normal level) (*n* = 42)	Children with MetS (monitoring/action level) (*n* = 18)	*p*-value^a^
Male, *n* (%)	30 (50)	23 (55)	7 (39)	0.06
Female, *n* (%)	30 (50)	19 (45)	11 (61)	0.08
Age, years, mean ± SD Median ± IQR	7.8 ± 2.4 9.0 ± 4.0	7.8 ± 2.4 9.0 ± 4.0	7.71 ± 2.4 8.5 ± 3.0	0.85
Weight, kg, mean ± SD	35.2 ± 14.1	35.2 ± 14.1	34.9 ± 14.1	0.004
Height, cm, mean ± SD	133 ± 16.9	133.36 ± 16.9	140.1 ± 13.6	0.22
BMI, mean ± SD Median ± IQR	19.0 ± 4.3 17.6 ± 6.7	19.0 ± 4.3 16.6 ± 4.8	18.8 ± 4.7 21.8 ± 4.9	0.001
Normal weight, *n* (%)	36 (60)	31 (74)	5 (28)	0.21
Overweight/obese, *n* (%)	24 (40)	11 (26)	13 (72)	0.002
WC, mean ± SD	67.0 ± 13.0	67.0 ± 13.0	13.0 ± 8.4	0.001
WHtR, mean ± SD	0.5 ± 0.1	0.5 ± 0.1	0.6 ± 0.1	0.001
Central obesity (WHtR ≥ 0.5), *n* (%)	32 (53)	16 (38)	16 (89)	0.001
PASI, mean ± SD Median ± IQR	4.3 ± 4.0 3.1 ± 3.0	4.3 ± 4.0 3.0 ± 3.0	4.0 ± 3.5 3.6 ± 3.6	0.54
Mild psoriasis, *n* (%)	42 (70)	29 (69)	13 (72)	0.14
Moderate-to-severe psoriasis, *n* (%)	18 (30)	13 (31)	5 (28)	0.21
Familial history of psoriasis, *n* (%)	34 (57)	24 (57)	10 (56)	0.44
Familial history of HPT and/or DM and/or HCT, *n* (%)	40 (67)	18 (45)	22 (55)	0.44

The mean age of children was 7.8 (± 2.4) years and 30 (50%) were boys. The mean value of BMI was 19.0 (± 4.3).

With respect to BMI, 36 (60%) children were normal weight (BMI < 85th and > 5th percentile), 12 (20%) children were overweight (≥ 85th and < 95th percentile), and 12 (20%) children were obese (≥ 95th percentile).

Thirty-two (53%) children had central obesity (WHtR ≥ 0.5).

The mean value of PASI was 4.3 (± 4.00); the mean value of BSA was 11.7 (± 5.5).

Children affected by moderate-to-severe psoriasis were 18 (30%). Thirty-four (57%) children had familial history of psoriasis and 40 (67%) children had familial history of hypertension and/or diabetes mellitus and/or hypercholesterolemia.

The mean value of fasting blood glucose (mg/dL) was 85.2 (± 8.1). The mean value of fasting blood insulin (mU/L) was 8.1 (± 6.7). The mean value of HDL-C (mmol/L) was 1.5 (± 0.4).

The mean value of TC (mmol/L) was 4.4 (± 0.9). The mean value of LDL-C (mmol/L) was 2.4 (± 0.7). The mean value of triglycerides (mg/dL) was 74.1 (± 53.1).

Eighteen (30%) children with psoriasis met the criteria for having MetS [16].

The prevalence of MetS in our children with psoriasis was significantly higher than the prevalence of MetS reported by the IDEFICS study in a general European pediatric (2–10 years) population (4.80%; *p* < 0.001).

Sixteen (27%) children had HOMA-IR ≥ 90th percentile. The same children also had HOMA 2-IR > 1.8.

The prevalence in our population of each metabolic altered parameter is shown in Table 2.

**Table 2:** Prevalence of altered metabolic parameters

	Patients (*n* = 60)
WC ≥ 90th percentile, *n* (%)	32 (53)
SBP ≥ 90th percentile or DBP ≥ 90th percentile, *n* (%)	8 (13)
HDL-C ≤ 10th percentile, *n* (%)	8 (13)
TC ≥ 90th percentile, *n* (%)	11 (18)
LDL-C ≥ 90th percentile, *n* (%)	7 (12)
Triglycerides ≥ 90th percentile, *n* (%)	7 (12)
Fasting blood glucose ≥ 90th percentile, *n* (%)	2 (3.3)
HOMA-IR ≥ 90th percentile and HOMA 2-IR > 1.8, *n* (%)	16 (27)

*SD* standard deviation, *IQR* interquartile range, *MetS* metabolic syndrome, *kg* kilograms, *cm* centimeters, *BMI* body mass index, *WC* waist circumference, *WHtR* waist-to-height ratio, *PASI* Psoriasis Area Severity Index, *HPT* hypertension, *DM* diabetes mellitus, *HCT* hypercholesterolemia, *SBP* systolic blood pressure, *DBP* diastolic blood pressure, *HDL-C* high-density lipoproteins-cholesterol, *TC* total cholesterol, *LDL-C* low-density lipoproteins-cholesterol, *HOMA-IR* homeostatic model assessment for insulin resistance, *HOMA 2-IR* homeostatic model assessment 2 for insulin resistance

^a^Comparison between children with and without MetS using χ^2^ test and Fisher’s exact test for the study of categorical variables and Student *t* test or Mann-Whitney nonparametric test for the study of quantitative variables

Children with MetS (“monitoring level” plus “action level”), compared with children without MetS (“normal level”), had significantly higher values of weight, BMI, WC, and WHtR and a higher prevalence of overweight/obesity and central obesity (respectively, *p* = 0.004; *p* < 0.001; *p* < 0.001; *p* < 0.001; *p* = 0.002; *p* < 0.001), but no statistically significant difference as regards duration of psoriasis, severity of psoriasis, and familial history of psoriasis (*p* > 0.1).

Multivariate logistic analysis showed that children with MetS, compared with children without, had a significantly higher value of WHtR and a significantly higher prevalence of overweight/obesity and familial history of hypertension (respectively, *p* = 0.04; *p* = 0.03; *p* = 0.02). The AUC of the fitted model was 0.83 and the Hosmer and Lemeshow goodness of fit test presented *p* > 0.1.

Children with HOMA-IR ≥ 90th percentile and HOMA 2-IR > 1.8 compared with children with HOMA-IR < 90th percentile and HOMA 2-IR ≤ 1.8 had a significantly higher values of BMI and WC and a higher prevalence of central obesity and overweight/obesity (respectively, *p* = 0.003; *p* < 0.001; *p* < 0.001; *p* < 0.001) (Table 1).

## Discussion

While several studies have reported that psoriasis is strongly associated with MetS in adults [1–4], limited data are currently available about the prevalence of MetS in childhood psoriasis [10–12].

Furthermore, while in adults a diagnosis of MetS is made when any three of the five risk factors are present (i.e., enlarged waist circumference with population-specific and country-specific criteria; elevated triglycerides, defined as ≥ 150 mg/dL; decreased HDL, defined as < 40 mg/dL in men and < 50 mg/dL in women; elevated blood pressure, defined as systolic blood pressure ≥ 130 mm Hg or diastolic blood pressure ≥ 85 mm Hg; and elevated fasting glucose, defined as blood glucose > 100 mg/dL) [24], there is actually no consensus about the definition of MetS in children and adolescents, mainly due to the lack of age-related reference values in pediatric age [16, 25, 26].

Recently, in 2014, however, a European population–based survey (the IDEFICS study) has estimated the prevalence of MetS using reference standards obtained in European children and has developed a quantitative MetS score, describing its distribution in children and making it possible to assess components of MetS in children [16].

Previously to the publication of the IDEFICS study, some authors had analyzed the prevalence of MetS in small groups of children with psoriasis.

Au et al. showed a higher prevalence of MetS in a group of 20 children (9–17 years old) with psoriasis, compared with an age- and gender-matched control group of 1.563 patients from the NHANES (National Health and Nutrition Examination Survey) (30% vs 7%) [10].

Goldminz et al. reported a prevalence of MetS of 30% in 20 subjects (aged 9–17 years; mean age 13.5 years; 75% female) with a current or previously documented history of psoriasis or arthritis, compared with a control group of age- and sex (but not ethnically)-matched children with nevi, warts, or acne, in which the prevalence of MetS was 5% [11].

Torres et al. observed a higher prevalence of MetS in 20 children aged 5–15 years with moderate-to-severe psoriasis, compared with a control group of 27 children with eczema, acne, or skin infections (25% vs 4%) [12].

These studies used the modified criteria of ATPIII proposed by de Ferranti et al. (20) which are referred to children aged 12–19 years and included both young children and adolescents (presumably pre- and post-pubertal children) [10–12].

As puberty incorporates various hormonal and body changes, including puberty-related accumulation of fat mass and reduced insulin sensitivity, we studied only pre-pubertal children [25, 27, 28].

In our pre-pubertal children with psoriasis, we found a prevalence of MetS significantly higher than the prevalence of MetS reported in a healthy European population of pre-pubertal children (2–10 years) (30% vs 5%) [16].

Furthermore, in our children with psoriasis, we found a statistically significant correlation between MetS and central obesity (*p* < 0.001) and overweight/obesity (*p* < 0.001).

This is consistent with the results of a recent study about metabolic status and its transition in children and adolescents which have identified children with central obesity as a “metabolic latent” group of patients which is likely to develop several MetS components over time [28].

This increased prevalence of MetS in our population of children with psoriasis is not surprising, as many metabolic parameters were altered in our patients.

As a matter of fact, in our study population, 24 (40%) children were overweight/obese, 32 (53%) children had central obesity, 8 (13%) children had high levels of blood pressure, 15 (25%) children showed low levels of HDL or high levels of triglycerides, and 2 (3%) children showed high levels of fasting blood glucose.

We also evaluated TC and LDL-C: 11 (18%) children had high levels of TC and 7 (12%) children had high levels of LDL-C (Table 2). These results were higher than data recently reported by Giussani et al. about the prevalence of high levels of TC (18% vs 4.5%) and LDL-C (12% vs 0.5%) in a large sample of Italian children [29].

Furthermore, in our population of children with psoriasis, 16 (27%) patients showed insulin resistance (HOMA-IR ≥ 90th percentile for age and sex and HOMA 2-IR > 1.8) [20, 22].

HOMA-IR seems to be a useful tool for an early evaluation of cardiometabolic risk in children and adolescents, independently of definition of MetS [30]. In fact, a recent systematic review by Arellano-Ruiz et al. indicated HOMA-IR as a reliable parameter to early identify patients which could have benefits of preventive and diagnostic therapeutic intervention [30].

Several studies have reported insulin resistance in adult patients with psoriasis [31–36], but, to the best of our knowledge, currently there are no previous studies of insulin resistance in large group of pre-pubertal children with psoriasis.

Of these 16 children with psoriasis and insulin resistance, all but 4 were overweight or obese (*n* = 12; 75%) and all but 2 had central obesity (*n* = 14; 88%), underlying once more the importance of evaluating WHtR in all children with psoriasis [6, 7].

A possible limitation of our study is the lack of an age- and sex-matched control group of healthy children from our area. But, in the same area, blood sampling in young healthy children is not routinely performed. On the other hand, patients with atopic dermatitis, acne, or other inflammatory skin diseases cannot be considered a healthy control group as these patients may have metabolic comorbidities [37, 38].

Therefore, we compared our data with the data on age- and sex-matched healthy children reported by the IDEFICS study and with the data reported in a large European group of children [16, 19].

## Conclusions

The high prevalence of MetS and of insulin resistance in our population of young children with psoriasis emphasizes the clinical relevance of assessing as soon as possible metabolic syndrome risk factors also in pre-pubertal children with psoriasis as they would probably benefit from lifestyle modifications in an effort to prevent cardiovascular diseases when adults.

## Supplementary Information

ESM 1 (DOCX 16 kb)

## Data Availability

n/a

## Abbreviations

BMI: Body mass index
BP: Blood pressure
BSA: Body surface area
HDL-C: High-density lipoprotein cholesterol
HOMA-IR: Homeostatic model assessment for insulin resistance
IDEFICS: Identification and prevention of Dietary- and lifestyle-induced health Effects in Children and infantS
LDL-C: Low-density lipoprotein cholesterol
MetS: Metabolic syndrome
PASI: Psoriasis Area Severity Index
TC: Total cholesterol
WC: Waist circumference
WHtR: Waist-to-height ratio
