# Supplementary material for: Metabolic syndrome and insulin resistance in pre-pubertal children with psoriasis
Source: Eur J Pediatr. 2021 Jan 22;180(6):1739–45. doi: 10.1007/s00431-020-03924-w (PMC8105196; doi:10.1007/s00431-020-03924-w)
Supplement: Supplementary file 1 — (DOCX 16 kb) [file 431_2020_3924_MOESM1_ESM.docx]

**Table:** results of multivariate analysis.

|  | **Regression**  **coefficient**  **(± SE)** | **Wald test**  **p-value** |
| --- | --- | --- |
| Over-weight/obesity | -1.52 (±0.75) | 0.03 |
| WHtR | 10.9 (±5.9) | 0.04 |
| Familial history of HPT | -1.55 (±0.75) | 0.02 |

SE=Standard Error. WHtR=Waist to Height Ratio. HPT=Hypertension.
